# Aflatoxin B_1_ Degradation by Ery4 Laccase: From In Vitro to Contaminated Corn

**DOI:** 10.3390/toxins15050310

**Published:** 2023-04-27

**Authors:** Martina Loi, Silvana De Leonardis, Biancamaria Ciasca, Costantino Paciolla, Giuseppina Mulè, Miriam Haidukowski

**Affiliations:** 1 Institute of Sciences of Food Production, National Research Council of Italy (CNR), Via Amendola 122/O, 70126 Bari, Italy; 2 Department of Biosciences, Biotechnology and Environment, Università degli Studi di Bari Aldo Moro, Via E. Orabona 4, 70125 Bari, Italy

**Keywords:** aflatoxin B_1_, laccase, corn, bioremediation, degradation products, hydrogen peroxide, ascorbic acid, dehydroascorbic acid, AFQ_1_, AFB_2a_, AFB_1_-diol

## Abstract

Aflatoxins (AFs) are toxic secondary metabolites produced by *Aspergillus* spp. and are found in food and feed as contaminants worldwide. Due to climate change, AFs occurrence is expected to increase also in western Europe. Therefore, to ensure food and feed safety, it is mandatory to develop green technologies for AFs reduction in contaminated matrices. With this regard, enzymatic degradation is an effective and environmentally friendly approach under mild operational conditions and with minor impact on the food and feed matrix. In this work, Ery4 laccase, acetosyringone, ascorbic acid, and dehydroascorbic acid were investigated in vitro, then applied in artificially contaminated corn for AFB_1_ reduction. AFB_1_ (0.1 µg/mL) was completely removed in vitro and reduced by 26% in corn. Several degradation products were detected in vitro by UHPLC-HRMS and likely corresponded to AFQ_1_, epi-AFQ_1_, AFB_1_-diol, or AFB_1_dialehyde, AFB_2a_, and AFM_1_. Protein content was not altered by the enzymatic treatment, while slightly higher levels of lipid peroxidation and H_2_O_2_ were detected. Although further studies are needed to improve AFB_1_ reduction and reduce the impact of this treatment in corn, the results of this study are promising and suggest that Ery4 laccase can be effectively applied for the reduction in AFB_1_ in corn.

## 1. Introduction

Aflatoxins (AFs) are secondary toxic metabolites produced by *Aspergillus* spp., which can contaminate food and feed worldwide [1]. AFs include more than 20 different furanocoumarin derivatives with carcinogenic, teratogenic, mutagenic, nephrotoxic, and hepatotoxic properties [2,3]. AFB_1_ is the most potent carcinogen known (Group 1 carcinogen) and the most occurring mycotoxin reported by the Rapid Alert System for Food and Feed [4]. AFs are chemically stable compounds, and currently their post-harvest reduction is performed only by physical methods, i.e., by sorting and adsorption. Thus far, effective AFs degradation can be achieved only by means of strong oxidants from physical (plasma, photolysis, photocatalysis), chemical (ammoniation), or biological (oxidoreductase enzymes) origin [5,6].

Enzymes represent an effective yet mild and environmentally friendly method to reduce AFs. So far, AFs enzymatic degradation has been achieved by using oxidoreductases, such as laccases, peroxidases, or so-called “aflatoxin oxidases” [7,8]. In particular, laccases (LCs, benzenediol: oxygen oxidoreductase, EC 1.10.3.2) are copper containing enzymes, able to oxidize simple and substituted phenolic compounds, thiols, anilines, amines, and complex aromatic compounds to the corresponding quinones, concurrently to the four-electron reduction in oxygen to water [9]. The catalytic activity of LCs can be further broadened to compounds which cannot be oxidized due to their high redox potential or steric hindrance thanks to the use of redox mediators. The use of these compounds allows for fine-tuning of the oxidation process and degrade a wide range of chemically unrelated compounds, such as mycotoxins [10]. Among redox mediators, the use of natural antioxidant compounds, such as phenols, has attracted attention because they are regarded as safe and can be used to improve existing industrial processes or develop new ones for the production of high value products [11]. Although the enzymatic degradation has been proven to be an effective method for mycotoxins reduction in feed, its application in food still has to be investigated. In Europe, Regulation 786/2015 defines the acceptability criteria for detoxification processes applied to products intended for animal feed [12].

A detoxification process implies that the toxin is converted to a less toxic, possibly safe, compound. Oxidases convert AFB_1_ into hydroxylated metabolite AFQ_1_, or to the 8,9-epoxy-AFB_1_, which spontaneously converts to 8,9-dihydroAFB_1_. Other hypothesized products derive from hydrolysis of the lactone ring followed by its opening (i.e., AFD_1_), from addition of water to the double bond of the terminal furan (AFB_2a_), or from demethylation (AFP_1_) [13]. These compounds have been found in vivo as a result of cytochrome detoxification in the liver [14].

Other than safety and efficacy, another mandatory requisite is that the method must not adversely affect the characteristics and the nature of the feed. Although food detoxification is not authorized yet, similar, if not more stringent, criteria will be likely set for food detoxification procedures in the near future.

Corn is one of the main staple food commodities worldwide and performs a central role in global agro-food systems. Contamination of corn grain with AFs is a concerning issue, especially in developing countries, where the majority of the product is self-produced by smallholder farmers in rural subsistence farming communities [15]. Despite being an important component of the human diet, corn is one of the main ingredients of livestock feed, it has multiple industrial uses, and its by-products find application in the energetic supply chain [16,17,18].

The application of an enzymatic degradation step within the common corn processing should encompass the addition of a buffered solution to easily convey the enzyme and natural redox mediators. Water addition is already included in both dry and wet milling processes.

Dry milling is the main industrial process used in the corn supply chain to separate the pericarp, the endosperm, and the germ; and obtain hominy grits, corn flours and feed meals [19,20]. It may encompass the tempering step, in which water is added to faster separate corn tissues and obtain fractions with low fat content, suitable for the manufacture of extruded products. In wet milling process, the kernels are steeped in SO_2_ and lactic acid solution for 24–48 h to facilitate the separation of kernel’s components [21]. In a complex matrix, such as corn flour, the addition of exogenous antioxidants could be investigated to support mediator reconversion and reduce the oxidative damage induced by the laccase mediator systems (LMS). Vitamin C (L-ascorbic acid, ascorbate, and ASC) is the most abundant water-soluble compound widely used as antioxidant in food and feed products. Its oxidation product, dehydroascorbic acid (DHA), in the apoplast, is readily taken up by the plasma membrane and reduced to ascorbate in the cytosol [22]. In this regard, either the direct or indirect (by reduced DHA) addition of vitamin C could be beneficial in supporting the enzymatic AFB_1_ reduction.

Therefore, in this work, an enzymatic treatment for AFB_1_ reduction was investigated in vitro using different LMSs, including acetosyringone (AS), a naturally occurring phenol, ASC, and DHA; in vitro degradation products were also identified. Then, AFB_1_ reduction was assayed in corn to assess enzyme performance in the real matrix. Additionally, to monitor the oxidative status, the effect of the different treatments in terms of protein content, lipid peroxidation, and H_2_O_2_ was also assessed.

## 2. Results

### 2.1. Aflatoxin B_1_ Degradation in Buffer Solution Using Different LMSs

In a previous work, the efficacy of different LMSs for AFB_1_ was screened in a 72 h-in vitr*o* assay. The maximum degradation of 1 µg/mL of toxin was 73%, obtained using AS as redox mediator [10]. This LMS was selected for further investigations to improve AFB_1_ degradation.

Therefore, in this study, AFB_1_ degradation (0.1 µg/mL) was evaluated over time using different LMS, namely Ery4 with AS, also in combination with ASC or DHA at 1 and 10 mM. Degradation, expressed as percentage with respect to the control not containing LC, is shown in Table 1.

AFB_1_ was completely removed from the buffer by Ery4 + AS even after only 1 h. The addition of ASC and DHA was deleterious, especially at higher concentrations. No degradation was observed using ASC. When used at 1 mM, DHA slowed the enzymatic degradation, and AFB_1_ was completely removed only after 24 h. DHA 10 mM inhibited AFB_1_ degradation, which reached only 20.3 ± 1.8% after 48 h.

### 2.2. In Vitro Study of Aflatoxin B_1_ Degradation Products

**Figure 1:**
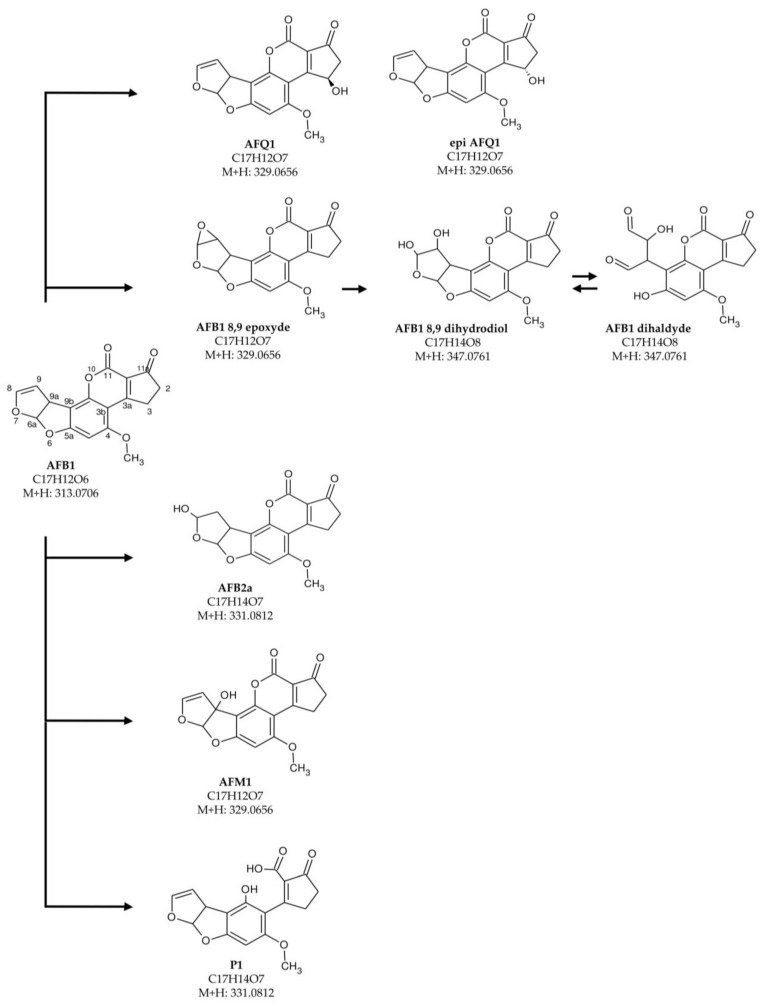
Proposed AFB_1_ degradation products.

To further study the ability of Ery 4 laccase to degrade AFB_1_ in the presence of the mediator AS, an UPLC-HRMS analysis was carried out. For this purpose, full-scan/variable data-independent acquisitions in positive ion mode of control samples containing Ery4 5 U/mL and AS 10 mM in sodium acetate buffer 1 mM (pH5) (C_Ery4_AS) and treated samples with AFB_1_ (1 µg/mL) incubated with Ery4 laccase (5 U/mL), and AS 10 mM in sodium acetate buffer 1 mM, pH 5, for 24 h (AF_Ery4_AS) were acquired. The comparison between the control and the AFB_1_-treated sample confirmed a decrease of 55% of AFB_1_ content and the formation of additional peaks after enzymatic treatment, which could be attributed to oxidation products of AFB_1_. Proposed reaction products, chemical structure and formulas are presented in Figure 1.

**Figure 2:**
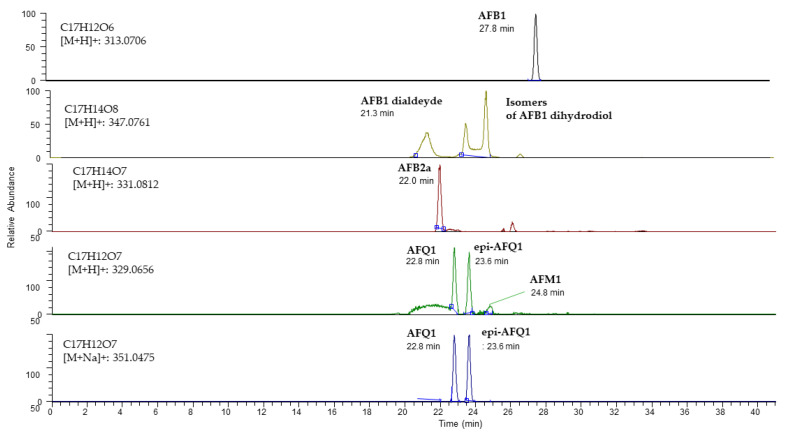
UHPLC-HRMS chromatogram of treated sample with AFB_1_ (1 µg/mL) incubated with Ery4 laccase (5 U/mL) and AS 10 mM in sodium acetate buffer 1 mM, pH5, for 24 h (AF_Ery4_AS). Peaks attributable to AFB1 and LMS oxidation products (AFB2a, AFQ_1_, epi AFQ_1_, AFM_1_, AFB_1_ dialdehyde and isomers of AFB_1_ dihydrodiol are shown. Resolution: 70,000 full width at half maximum; extraction window tolerance 5 ppm.

A measured mass of 347.0761, which was attributable to a molecular formula C_17_H_14_O_8_ corresponding to the ion [M+H]^+^, showed one peak eluting at 21.5 min (mass error: 1.6 ppm) and two overlapping peaks at 23.6 min (mass error: 1.3 ppm) and 24.7 min (mass error: 1.3 min) (Figure 2). A difference of 34 mass units compared to aflatoxin B_1_ indicated the presence of two hydroxyl groups; therefore, the following molecular formula could be attributed to AFB_1_ 8,9-dihydrodiol or to AFB_1_ dialdehyde. Considering the polarity of these compounds, the peak at 21.5 was assumed to be relative to dihydrodiol or dialdehyde. The [M+H]^+^ molecular ion at 331.0812, which was attributable to a molecular formula C_17_H_14_O_7_, showed one main peak at 22.0 min (mass error: 1.2 ppm) and could be related to AFB_2a_ or product 1 (P1) (Figure 1). Finally, the [M+H]^+^ molecular ion at 329.0656, which was attributable to a molecular formula of C_17_H_13_O_7_, corresponded to two main peaks, eluting at 22.8 min (mass error: 2.2 ppm) and 23.6 min (mass error: 1.9 ppm); one less abundant peak eluted at 24.8 min (mass error: 3.2 ppm). These peaks could be related to AFQ_1_, epi AFQ_1_, AFB_1_-8,9-epoxyde, or AFM_1_.

**Figure 3:**
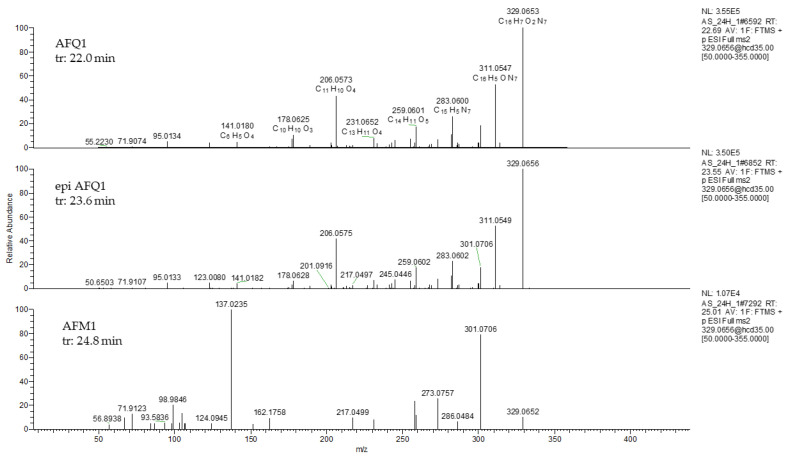
Parallel reaction monitoring (PRM) spectra (collision energy 35 eV) of 329.0656 in treated sample with AFB1 (1 µg/mL) incubated with Ery4 laccase (5 U/mL) and AS 10 mM in sodium acetate buffer 1 mM, pH 5, for 24 h (AF_Ery4_AS).

Identity confirmation of the putative product of the enzymatic reaction was performed by matching the detected fragments with MS2 spectra reported in the literature (if available), as shown in Table 2. In the case of precursor at 329.0656, fragments obtained in AF_Ery4_AS sample were reported in Figure 3. MS/MS spectra of the first two peaks (22.8 min and 23.6 min) showed some characteristic fragments of AFQ_1_, such as the peak of m/z 311.0547, originated by the loss of water (neutral loss of 18 a.m.u.), and fragments of m/z 283.0606, 206.0673, and 141.0180.

Peak eluting at 24.81 min presented different relative abundances of fragments 329.0652 and 301.0706. In addition, the fragment ion at 273.0757 [M − 74 + H]^+^ was shown. These fragments are characteristic of AFM_1_ [9,23].

In the case of the precursor at 331.0812, fragments at m/z 303.0861 [M-CO + H]^+^, 284.0316, 267.0288, and 239.0338 were shown. No fragments were detected for the precursor at 347.0812.

A rough estimation on the basis of peak area ratios indicated that among the identified products, the most prevalent one was AFQ_1_ (41.2%), followed by AFB_2a_/P1 (29.6%), AFB_1_-dihydrodiol/AFB_1_dialdehyde (14.8%), and AFM_1_ (3.7%). AFB_2a_ may also be formed spontaneously in acidic conditions, in agreement with other literature data [13].

### 2.3. Aflatoxin B_1_ Degradation in Corn

**Figure 4:**
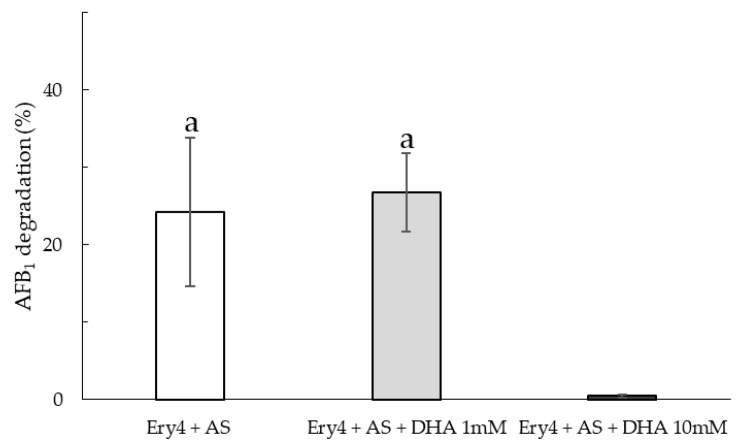
Aflatoxin B_1_ degradation (%) in corn samples treated with Ery4 (5 U/mL) acetosyringone (AS, 10 mM) 10 mM and dehydroascorbic acid (DHA) at 1 and 10 mM. Different lowercase letters above columns indicate significant differences between treatments (*p* < 0.05).

Following the results obtained in vitro, only three LMSs (Ery4, AS and DHA) were tested in artificially contaminated corn flour (50 µg/kg AFB_1_). After the reaction, samples were centrifuged, and both the supernatant and pellets were analyzed. No AFB_1_ was detected in the supernatant, while appreciable degradation could be observed in the pellets (Figure 4). AFB_1_ degradation levels were lower with respect to the **in vitro** trials, although Ery4+AS was confirmed to be the most efficient LMS. While no difference could be observed when DHA 1 mM was added, a clear inhibiting effect was exerted by DHA 10 mM, leading to ineffective degradation.

### 2.4. Protein Content

**Figure 5:**
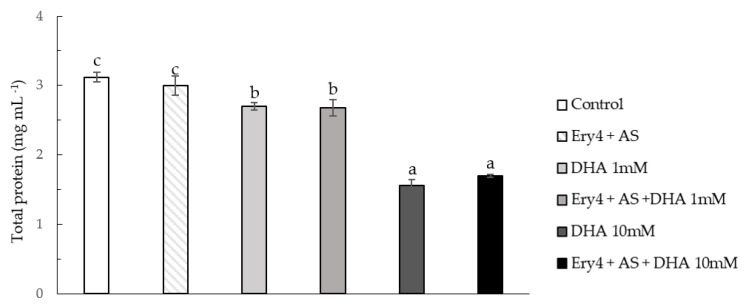
Total protein content (mg mL^−1^) in untreated corn samples (Control) and samples treated with Ery4 (5 U/mL), acetosyringone (AS, 10 mM), and dehydroascorbic acid (DHA) at 1 and 10 mM. Different lowercase letters above columns indicate significant differences between treatments (*p* < 0.05).

As shown in Figure 5, the enzymatic treatment did not alter the total protein content, calculated as a sum of water-soluble, ethanol soluble, and insoluble fractions. Conversely, statistically significant differences were shown in samples containing DHA. In particular, a dose dependent reduction was observed irrespectively of the presence of Ery4 and AS, highlighting that protein reduction could be ascribed to DHA addition rather than to LMS.

### 2.5. Lipid Peroxidation and H_2_O_2_ Content

**Figure 6:**
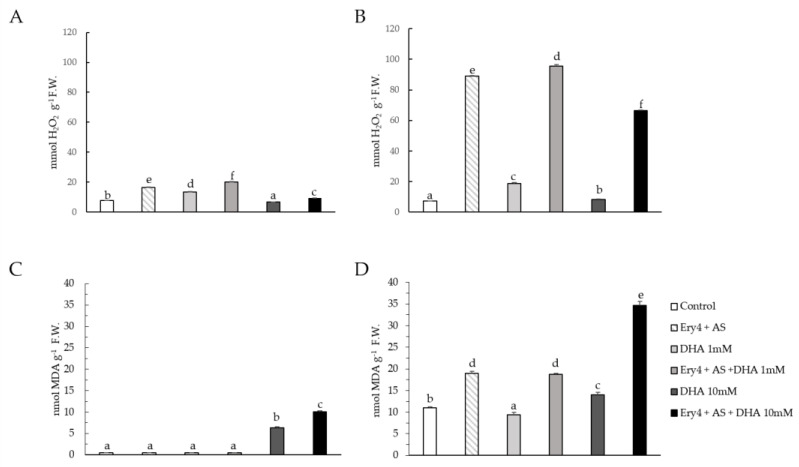
Hydrogen peroxide (Panel (**A**), pellet; Panel (**B**), supernatant) and lipid peroxidation (Panel (**C**), pellet; Panel (**D**), supernatant) content of corn samples using Ery4 (5 U/mL) acetosyringone (AS) 10 mM, and dehydroascorbic acid (DHA) at 1 and 10 mM. Data were expressed as mmol or nmol per fresh weight (F.W.). Different lowercase letters above columns indicate significant differences between treatments (*p* < 0.05).

The oxidative status of both supernatant and pellets was analyzed in terms of H_2_O_2_ content and MDA levels. The enzymatic treatment had a detrimental effect on H_2_O_2_ content, both in the pellet (Figure 6A) and in the supernatant (Figure 6B; a synergistic oxidative effect was observed in samples treated with DHA 1 mM, as H_2_O_2_ levels further increased up to 95,65 ± 0.79 mmol/mL. Conversely, lower H_2_O_2_ values were registered in samples containing DHA 10 mM (66.5 ± 0.20 mmol/mL).

As reported for H_2_O_2_, higher MDA content was shown in the supernatants rather than in the pellets. In this latter case, only samples containing DHA 10 mM showed statistically significant increased level. In the supernatants, the oxidative effect of Ery4 + AS enzymatic treatment was more pronounced, and the synergic effect of DHA could be observed only at 10 mM concentration.

## 3. Discussion

Mycotoxins degradation via LMS has been explored with several mediators of natural and synthetic origin [10]. Natural phenols, such as AS, were applied as promising mediators for bioremediation, with potential application in the food industry [24].

AS is a syringic acid derivative found as phenolic humic constituents in natural organic matter [25]. AS, together with other structurally related compounds, have been reported to be efficient mediators for the reduction in organic pollutants, dyes, and mycotoxins [10,26,27,28].

AS has a redox potential of 0.580 V, which is not among the highest potential reported for LC mediators. Nonetheless, the mediator efficacy does not only depend upon the redox potential but also on the rate of oxidation by LC, stability of the oxidized form of the mediator, its capacity of being recycled, and not to inhibit LC active site [29]. AS’s good mediator activity is due to the presence of 2,6-dimethoxy electron-donating groups that give stable phenoxy radicals with a relative long half-life and low free radical activity [30,31].

AS oxidation was reported to proceed via electron transfer and hydrogen atom abstraction mechanism to give a phenoxy radical. This radical intermediate is also stabilized by the acetyl group in orto position, where a further electron delocalization takes place. Additionally, AS oxidation intermediates can still be oxidized by LC as long as it has a phenolic group that can be oxidized [30]. Due to the radical nature of the oxidation mechanism, the addition of a natural antioxidant, ASC, was evaluated for AS reconversion. Moreover, due to the existing reconversion route of ASC from DHA in plasma membrane, DHA supplementation was also assayed.

ASC is a pivotal antioxidant compound and a key element for the metabolism of almost all living organisms. It is a dibasic acid with an enediol group on C2 and C3 of a heterocyclic lactone ring, and at physiological pH, the hydroxyl group at C3 is deprotonated, giving a monovalent anion, ASC [32,33].

The ASC is the only reductant present at a significant level in the apoplast, with a redox potential ranging from +0.40 to +0.50 V [34]. When both electrons of the enediol group of ASC are donated, ASC can be oxidized in this compartment to DHA by ASC oxidases [22,35]. Conversely, when in excess, DHA can be transported via the cell membrane through a carrier mediated uptake and reduced again to ASC. This is part of the cellular redox gradient across the plasma membrane, connecting intra- and extra-cellular environments. The redox environment of the cell is determined by the global poise of its oxidation/reduction systems and may contribute to regulating the effectiveness of the LMS. Indeed, there is a complex link between redox state and simplistic and apoplastic metabolism [36], which is also determined by ROS level, produced at either physiological or toxic levels [35].

The addition of ASC completely inhibited AFB_1_ degradation. Similarly, DHA negatively impacted AFB_1_ degradation, proving that DHA does not participate in LMS and possibly inhibits LC at high concentration.

ASC was reported to non-competitively inhibit LC from *Botritis cynerea* [37]. Accordingly, in our study, ASC reduced the rate of AFB_1_ degradation, possibly by inhibiting LC or scavenging AS reactive radicals before toxin degradation. To our knowledge, no report is available on how DHA affects LC activity. DHA can undergo further irreversible degradation, such as hydrolyzation to 2,3-diketo-L-gulonate, or oxidation to a range of products, such as L-threonic acid, oxalic acid, and their esters; therefore, it may contribute to radical quencing [38].

In the present study, we wanted also to investigate the effectiveness of the detoxification process of AFB_1_ by LMS under the optimal degradation condition. To this purpose, a UHPLC-HRMS analysis was carried out to investigate the degradation products by LMS in presence of AS 10 mM after 24 h incubation and 1 µg/mL in **in vitro** samples. To date, neither the mechanism of the laccase-catalyzed degradation of AFB_1_ nor the degradation products have been fully disclosed; however, a review on the application of both bacterial and fungal laccase enzyme in AFB_1_ degradation was reported by Okawara and colleagues [39]. LCs act on AFB_1_ in two ways; on the terminal furan ring of AFB_1_, leading to the formation of AFB_1_-8,9 epoxide, which is further converted to AFB_1_-8,9 dihydrodiol or may directly open the lactone ring by introducing hydroxyl groups at the carbon 10 and 11 positions in AFB_1_ (product P1 Figure 1).

*Trametes versicolor* laccase, Lac2 from *Pleurotus pulmonarius* and the Ery4 from *P. eryng*ii were demonstrated to degrade AFB_1_ via the mediation of natural phenolic compounds such as AS, syringaldehyde, ferulic acid, etc., or synthetic compounds; however, the degradation products have not been reported. The oxidation of AFB_1_ in AFQ_1_ was reported in degradation study on CotA laccase from *Bacillus licheniformis* [7] and on Lac2 produced by *Cerrena unicolor* 6884 [40]. The latter study also reported the presence of AFQ_1_ epimer (epi AFQ_1_). In these studies, the presence of AFQ_1_ and epi AFQ_1_ products was justified assuming the action of LMS on the lactone ring of AFB_1_, possibly by hydrogen atom transfer followed by addition of water to C3. In our study, based on the structure, relative polarity and fragment ions, the epi AFQ_1_, AFQ_1_, and AFM_1_ were identified as oxidation products, corresponding to the peaks at 22.8, 23.6, and 24.8 min. The presence of AFQ_1_ is in agreement with the several reports of AFB_1_ degradation with LCs [40,41], peroxidases [8], or other oxidases [7].

LC and oxidases were also reported to convert AFB_1_ into the toxic 8,9-AFB_1_ epoxide [13,39]. Nonetheless, this compound has a fast rate, non-enzymatic conversion to AFB_1_-diol in water [42], thus it is hardly detectable by UPLC-HRMS. Based on 8,9-AFB_1_ epoxide hydrolysis and kinetics of rearrangement of the dihydrodiol, and considering LMS mechanism, retention times, and polarity of detected compounds in UPLC-HRMS, the ion at m/z 347.0812 corresponding to a molecular formula C_17_H_14_O_8_ could be likely addressed as AFB_1_-dialdehyde or AFB_1_-diol isomers.

In addition, a measured mass of m/z 331.0812, which was attributable to the molecular formula C_17_H_14_O_7_, corresponding to the ion [M+H]^+^, with a mass accuracy of 1.2 ppm, was detected. Two candidate compounds were in agreement with this formula (AFB_2a_ and P1). Despite LCs have been reported to degrade AFB_1_ into P_1_, according to polarity compounds, the measured mass of m/z 331.0812 at retention time 22.0 min could be more likely AFB_2a_.

All detected degradation products show a higher polarity and a higher excretion rate via urine and faeces, thus, lower toxicity than AFB_1_ [43]. Some of the products found lack of the reactive C8–C9 double bond and possess reduced mutagenicity. Nonetheless, they retain the ability to form Shiff bases with primary amines in proteins, leading to adducts responsible for residual cytotoxicity.

The same degradation trend was registered in vitro and in corn samples, though the differences were evened out, likely due to the matrix effect. Indeed, the interaction between the toxin and the active mediator may be hindered by proteins, carbohydrates, and lipids in corn flour, resulting in a lower efficacy. Competition of food components for the enzyme, enzyme adsorption to food components, and higher viscosity may also contribute to reducing the efficacy of LMS in corn flour.

The enzymatic treatment had slightly impacted the oxidative status of the matrix, while more significant effects were observed in the supernatants. The increased MDA content in corn sample pellets treated with higher DHA-concentration indicated the presence of increased lipid peroxidation of the biological membranes. This reflects the fact that the DHA is toxic in cell if is largely accumulated [44] and may activate induced systemic resistance via ROS production and salicylic acid pathway activation [45]. On the other hand, the decreased hydrogen peroxide level observed, at least for the corn pellet samples treated with the highest DHA concentration, indicates that H_2_O_2_ could oxidize the biological membranes, as supported by the higher lipid peroxidation in these samples.

Overall, these results suggest that the transport mechanism for DHA via the plasma membrane with its reconversion to ASC would appear not to be present, at least for the corn kernel.

H_2_O_2_ is the most commonly studied ROS due to its stability and capability of penetrate through cellular membranes, and it has been recognized as a subcellular signaling molecule. Plants can well tolerate relatively high H_2_O_2_ (up to 10^2^–2 × 10^5^ μM), and its endogenous concentration was reported to range from nanomoles to several hundred micromoles [46,47]. Thus, H_2_O_2_ levels found in this study, although significantly higher in the LMS treated samples, were still in the tolerable ranges reported in the literature for plant cells [47].

The decreased total protein content in the DHA-treated samples underlines the presence of an action of DHA on the protein structure and an interference with the dye response. Particularly, corn proteins are rich in prolamins, which are thiol containing proteins. Indeed, a link between reduction DHA and oxidation of thiol group has been found [48]. Consequently, this event could have a negative impact on the protein folding due to the interaction of carbonyl groups of the DHA with amino acid residues. Indeed, DHA irreversibly inhibits some enzymes, such as human type I hexokinase, that shows a smaller number of cysteine residues [49,50].

A wide number of reports of in vitro enzymatic AFs degradation are available in the literature [10,51,52]. Conversely, fewer studies have been conducted on food matrices. Enzymatic degradation has been explored in food or feed for AFs, zearalenone, thricothecenes, and fumonisin [53,54,55,56], although they did not focus on the evaluation of the characteristics of the food matrix after the treatment. To our knowledge, this is the first study that evaluates the oxidative status of corn flour after the application of an enzyme-based degradation treatment.

In order to be applied in feed matrices, mycotoxin reduction methods must not alter the characteristics of the matrix. Therefore, the evaluation of the effects exerted by any reduction treatment must be assessed. So far, this is the first time that the effects on protein content and the oxidative status of corn flour after an enzymatic reduction treatment were studied. Indeed, the work performed by Dini and colleagues [57] only focus on aflatoxins enzymatic degradation in corn flour, obtaining similar results (30% of reduction) with the same level of contamination. Aflatoxin degradation was studied in other matrices, such as milk and beer, with promising results [8,10,58,59,60,61]. Nonetheless, few studies investigated the effect of the enzymatic treatment on the protein content and quality, antioxidant activity and technological properties though in a liquid matrix, such as milk [60,61].

## 4. Conclusions

Different LMSs were tested in vitro and in corn flour with the aim of reducing AFB_1_ contamination. Complete degradation was achieved in vitro with Ery4 and AS; the addition of ASC completely inhibits the degradation, while DHA decreased AFB_1_ degradation in a dose-dependent manner. The same behavior was observed in corn, even though the rate of degradation was reduced of one fourth due to matrix effect. Several degradation products characterized by lower toxicity were found in vitro by UHPLC-HRMS, namely AFQ_1_, epi-AFQ_1_, AFB_1_-diol or AFB_1_dialehyde, AFB_2a_, and AFM_1_.

The protein content was not altered by the sole enzymatic treatment, while it was lowered by DHA in a dose dependent manner. Conversely, LMS treatment affected the oxidative status of corn flour. Increased lipid peroxidation and H_2_O_2_ content were registered in enzyme- treated samples irrespectively of the amount of DHA added.

Even though further studies are needed to reduce matrix effect and assess the technological impact of this reduction methods, the results of this study are promising and suggest that AFB_1_ can be reduced completely in vitro and by 26% in corn flour. Therefore, since only slight oxidation occurred in corn flour, minimum impairment of the nutritional or technological properties could be expected by this treatment, but with significant improvement in its safety.

## 5. Materials and Methods

### 5.1. Chemicals, Reagents, and Corn Kernels

Analytical-grade acetonitrile (ACN), methanol (MeOH), and toluene (for HPLC purpose) were purchased from Mallinckrodt Baker (Milan, Italy). Ultrapure water was produced by a Millipore Milli-Q system (Millipore, Bedford, MA, USA). Filter paper and Glass microfiber filters (GF/A) were purchased from Whatman (Maidstone, UK).

Standard of aflatoxin B_1_, 2-azino-di-[3-ethylbenzo-thiazolin-sulphonate] (ABTS), syringaldehyde, and acetosyringone were obtained from Sigma Aldrich (Milan, Italy). Immunoaffinity columns AflaTest^®^ Wide Bore were obtained from Vicam L.P. (Watertown, MA, USA).

Organic corn kernels (*Zea Mais* L.) were purchased from Bioseme s.c.a.r.l.

### 5.2. Preparation of Standards

Standard solution of AFB_1_ was prepared by dissolving the solid commercial toxin in toluene/acetonitrile (9:1, *v*/*v*) to a concentration of 10 µg/mL. The exact concentration of AFB_1_ was determined according to AOAC Official Method 971.22 [62]. Aliquots of the solution were transferred to 4 mL amber silanized glass vials and evaporated to dryness under a stream of nitrogen at 50 °C. The residue was dissolved with water/methanol (60:40, *v*/*v*) to obtain final concentrations in a range of 0.5 to 50 ng/mL of aflatoxin B_1_. Standard solutions were stored at −20 °C and warmed to room temperature before use.

### 5.3. Laccase Production and Purification

The recombinant Ery4 laccase was produced from *Saccharomyces cerevisiae* ITEM 17,289 of the Agri-Food Microbial Fungi Culture Collection of the Institute of Sciences of Food (http://www.ispa.cnr.it/Collection, accessed on 25 October 2022). Laccase purification was performed by concentration/ultrafiltration of the cultured media with Tris HCl 50 mM, pH 8, and anion exchange chromatography, as reported in Loi et al. [61].

### 5.4. Laccase Activity Assay

The enzymatic activity was assessed by the ABTS colorimetric assay using a spectrophotometer (Ultraspec 3100pro, Amersham Pharmacia Biotech Italia, Cologno Monzese, Italy). [7]. The reaction was performed in 100 mM sodium acetate pH 4.5, 5 mM ABTS and an appropriate amount of enzyme solution in a final volume of 1 mL. The oxidation of ABTS was determined after 10 min at 420 nm (ε420 = 36,000 M^−1^cm^−1^). One unit was defined as the amount of enzyme which oxidized 1 µmol of substrate per min.

### 5.5. Aflatoxin B_1_ Degradation In Vitro

AFB_1_ degradation (0.1 µg/mL) was assessed in sodium acetate buffer (1 mM, pH 5) using 5 U/mL of Ery4 laccase and AS 10 mM. ASC and DHA were also tested at two concentrations (1 or 10 mM). Aliquots were incubated at 25 °C and withdrawn after 1 h, 2 h, 3 h,6 h, 24 h, and 48 h, respectively, then immediately added with methanol (1:1 *v*/*v*) and stored at −20 °C until analysis.

### 5.6. In Vitro Study of Aflatoxin B_1_ Degradation Products

In order to analyze AFB_1_ degradation products, a degradation assay was performed as described in Section 5.4, but with higher amount of toxin (1 µg/mL). Controls and samples containing Ery4 were analyzed after 24 h of static incubation at 25 °C.

### 5.7. UHPLC-HRMS Analysis

The UHPLC-HMRS analysis was performed on a Q-Exactive Plus mass spectrometer equipped with a heated electrospray ion source (HESI II) coupled to an Ultimate 3000 UHPLC system (all from Thermo Fisher Scientific, San Jose, CA, USA).

The LC column was a Gemini C18 column (150 mm × 2 mm, 5-µm particles) (Phenomenex, Torrance, CA, USA) preceded by a Gemini C18 guard column (4 mm × 2 mm). The mass spectrometer operated in full scan mode combined with 5 MS2 events (all related instrumental parameters can be found in Ciasca et al. (2020) [63]. In addition, putative compound was identified by target MS/MS analysis (parallel reaction monitoring (PRM) mode). Settings for PRM data acquisition were as follows: resolution, 70,000 fwhm; microscans, 1; AGC target, 5 × 10^5^; maximum injection time, 200 ms; isolation window, 0.5 m/z; nor-malized collision energy (NCE), 35 eV; spectrum data type, centroid. The inclusion list contained the monoisotopic masses of main significant features. The system was controlled by the Xcalibur (version 3.1), Chromeleon MS Link 6.8, and Q-Exactive Tune 2.8 software package.

### 5.8. Aflatoxin B_1_ Degradation in Corn

Corn kernels were finely ground (≤500 µm of diameter) by a Model Retsch ZM 200 laboratory mill (Retsch, Haan, Germany) and spiked with 50 µg/kg of AFB_1_. The sample was left all night to allow solvent evaporation prior to perform the degradation test.

The enzymatic reactions were performed using 2 g of corn flour in 15 mL tubes with 6 mL of sodium acetate buffer containing Ery4 (5 U/mL) and AS 10 mM. The effect of DHA was also evaluated together with Ery4 and AS at two different concentrations, namely 1 and 10 mM. Samples were incubated at 25 °C under shaking 150 rpm for 3 h.

### 5.9. Aflatoxin Extraction and Chemical Analyses

#### 5.9.1. Corn Samples Clean-Up

After incubation, all sample tubes were centrifuged at 15,000 rpm for 10 min, giving a supernatant (buffer) and a pellet (corn flour); then, AFB_1_ was quantified. AFB_1_ analyses were performed according to the AOAC Official Method 991.31 [64], based on immunoaffinity column clean-up and toxin determination by HPLC/FLD with post-column photochemical derivatization (UVE™, LCTech GmbH, Dorfen, Germany).

Briefly, the pellet plus 0.5 g of NaCl was extracted with 8 mL of methanol/water (70:30, *v*/*v*) by 60 min shaking. After filtration (filter paper, Whatman n. 4), 4 mL was diluted with 8 mL water and filter (glass microfiber filter, Whatman GF/A). The supernatant was filter through glass microfiber filter. A total of 6 mL of pellet extract fraction and 3 mL supernatant extract were purified through Afla Test™ WB immunoaffinity column. The column was washed with 10 mL water, then eluted with 1 mL methanol. Afterwards the extracts were diluted with 1 mL of water.

#### 5.9.2. HPLC Analyses

Analyses were performed on a HPLC apparatus with a full loop injection system; 100 µL of each sample were injected. The fluorometric detector was set at wavelengths of 365 nm (excitation) and 435 nm (emission). The mobile phase consisted of a mixture of water/acetonitrile (70:30, *v*/*v*), and the flow rate was 1.0 mL/min. The temperature of the column was maintained at 40 °C. AFB_1_ was quantified by measuring peak areas at the retention time of aflatoxin standard solutions and comparing these areas with the relevant calibration curve. With this mobile phase, the retention time was about 12 min. The limit of quantification (LOQ) was 2 µg/kg for pellet and 1 µg/kg for supernatant based on a signal to noise ratio of 10:1, and the limit of detection (LOD) were 1 µg/kg for pellet and 0.5 µg/kg for supernatant based on a signal to noise ratio of 3:1.

### 5.10. Lipid Peroxidation and H_2_O_2_ Content

Lipid peroxidation was measured in terms of malondialdehyde (MDA) concentrations, following the method reported by Villani and colleagues [65]. Absorbance was measured at 532 and 600 nm, and MDA content was calculated and expressed as nmol g^−1^ fresh weight.

The homogenate was filtered through four layers of cheesecloth to remove cellular debris and then centrifuged at 18,000× *g* for 20 min at 4 °C. The H_2_O_2_ content was measured as reported by Lanubile et al. [66]. A supernatant aliquot of the reaction mixture was read at 436 nm, and its absorbance was compared to the extinction coefficient of an H_2_O_2_ standard.

### 5.11. Protein Content

After the enzymatic treatment, samples were added with NaCl 0.4 M and 0.4% (*v*:*v*) of protease inhibitor cocktail (Sigma Aldrich, Milan, Italy) and incubated for additional 20 min. Then, samples were centrifuged at 10,000 rpm for 20 min, and the pellet and supernatant were separated.

The supernatant was dialyzed against H_2_O for 3 h to obtain the first water-soluble protein fraction. The pellet was resuspended in a solution containing EtOH 70% and 2-mercaptoethanol 0.01 M and incubated for 20 min. After centrifugation at 10,000 rpm for 20 min, an ethanol soluble fraction was obtained, while the pellet was further extracted using PBS 0.1 M, pH 7.4, SDS 2.5%, and NaCl 0.01 M to obtain the alcohol-insoluble protein fraction. The three protein fractions were quantified using Bradford method [67].

### 5.12. Statistical Analyses

Data are the means ± standard deviation of at least three independent biological replicates. One-factor analysis of variance (ANOVA), followed by Tukey’s HSD test, was performed on means. Differences between samples and relative control were considered significant for a *p* < 0.05.

## Figures and Tables

**Table 1:** Time course in vitro degradation of aflatoxin B_1_ (0.1 µg/mL) using Ery4 laccase (5 U/mL), acetosyringone (AS) in combination with dehydroascorbic acid (DHA) 1 or 10 mM.

Time (h)	Ery4 + AS	Ery4 + AS + DHA 1 mM	Ery4 + AS + DHA 10 mM
1	100	84.1 ± 3.5	6.1 ± 1.0
2	100	90.3 ± 0.8	8.6 ± 6.5
3	100	93.2 ± 2.7	8.4 ± 4.2
6	100	95.7 ± 1.9	11.8 ± 0.3
24	100	100	16.6 ± 1.6
48	100	100	20.3 ± 1.8

**Table 2:** Precursor ion, exact mass, retention time, and fragments of proposed AFB_1_ degradation products.

Proposed Product	Molecular Formula	Mass Exact [M+H]^+^/[M+Na]^+^	Error (ppm)	Retention Time (min)	Fragments
AFB_1_-dialdehyde/AFB_1_-dihydrodiol	C_17_H_14_O_8_	347.0761	1.6	21.3	No data
AFB_2a_/P1	C_17_H_14_O_7_	331.0812	1.2	22.0	303.0861, 299.0550, 284.0316, 267.0288, 239.0338
AFQ_1_	C_17_H_12_O_7_	329.0656/351.0475	2.2	22.8	311.0343, 283.0601, 259.0601, 247.0602
Unidentified peak 1	C_17_H_14_O_8_	347.0761	1.3	23.6	No data
epi-AFQ_1_	C_17_H_12_O_7_	329.0656/351.0475	1.9	23.6	311.0343, 283.0601, 259.0601, 247.0603
Unidentified peak 2	C_17_H_14_O_8_	347.0761	1.3	24.7	No data
AFM_1_	C_17_H_12_O_7_	329.0656/351.0475	3.2	24.8	329.0656, 301.07, 273.05
Unidentified peak3	C_17_H_14_O_8_	347.0761	2.3	26.7	No data
AFB_1_	C_17_H_12_O_6_	313.0706	1.0	27.8	285.0575, 270.0522, 243.0652, 201.0912

## Data Availability

Not applicable.
